# Acute Suffering, Act of Togetherness and On‐Scene Space: A Concept Synthesis for Caring in Prehospital Emergency Care

**DOI:** 10.1111/scs.70335

**Published:** 2026-11-10

**Authors:** Mats Holmberg, Eivor Wallinvirta, Andreas Rantala, Anders Bremer

**Affiliations:** ^1^ Department of Health Sciences, Innovation and Design, Faculty of Engineering and Health Sciences Mälardalen University Eskilstuna Sweden; ^2^ Department of Health and Caring Sciences, Faculty of Health and Life Sciences Linnaeus University Växjö Sweden; ^3^ Centre of Interprofessional Collaboration Within Emergency Care (CICE), Linnaeus University Växjö Sweden; ^4^ Department of Ambulance Service Region Sörmland Katrineholm Sweden; ^5^ Research and Development Arcada University of Applied Sciences Helsinki Finland; ^6^ Department of Health Sciences Lund University Lund Sweden; ^7^ Department of Ambulance Service Office of Medical Services, Region Skåne Helsingborg Sweden; ^8^ Department of Ambulance Service Region Kalmar County Kalmar Sweden

**Keywords:** acute suffering, caring science, concept synthesis, on‐scene space, prehospital emergency care, togetherness

## Abstract

**Background:**

Prehospital emergency care attends to patients experiencing acute suffering; a sudden and profound disruption affecting the person's life, body and existential well‐being in the context of illness, injury or crisis. In this unpredictable and vulnerable setting, a central question emerges: how can caring needs be effectively met?

**Aim:**

The aim of this paper is to develop a concept synthesis grounded in a caring science paradigm within the prehospital context, clarifying the nature of caring.

**Method:**

A concept synthesis based on a caring science theoretical framework was conducted using a combined deductive–inductive approach, integrating theory, clinical experience and relevant literature through an abductive and iterative process.

**Results:**

The synthesis introduces three interrelated concepts: acute Suffering (S), act of Togetherness (T) and On‐scene space (O), situated within Prehospital emergency care (P). These concepts collectively shape the STOP synthesis. STOP positions caring as the core of prehospital emergency care, emphasizing a holistic perspective that extends beyond a purely biological understanding. It provides a structured framework for understanding and practicing the complexity of caring in this setting.

**Conclusion:**

Using the STOP‐sign metaphor, the STOP synthesis highlights the importance of pausing in prehospital emergency care to recognize the patient as a multidimensional human being experiencing acute suffering. By integrating the concepts of acute Suffering, act of Togetherness and On‐scene space within the prehospital context, the synthesis promotes a shift beyond a solely biomedical perspective toward a caring science understanding of suffering and care. The synthesis may support prehospital professionals, particularly nurses, in integrating caring and medical knowledge in clinical practice and provides a theoretical basis for future research, education and a holistic prehospital emergency care.

## 1 Introduction

Prehospital emergency care is provided primarily by ambulance services, forming a central component of Emergency Medical Services (EMS). This context differs from many other settings as care is mobile and often delivered in patients' homes or public spaces, with limited prior information. The presence of bystanders places added demands on maintaining privacy and integrity, while small teams require rapid, independent assessment and decision‐making. EMS provides urgent care for trauma, life‐threatening conditions and non‐critical emergencies and has traditionally been shaped by emergency medicine, traumatology and disaster medicine. Internationally, EMS staff commonly include paramedics and emergency medical technicians (EMTs). In contrast, Sweden and Finland rely on advanced life support units staffed mainly by dyadic teams of two registered nurses (RNs) or one RN with an EMT/paramedic [1]. In Sweden, RNs constitute 70%–80% of the EMS workforce, with the remaining positions filled by EMTs/paramedics with varying educational backgrounds [2].

Here, ambulance healthcare professionals refer collectively to paramedics, EMTs/paramedics, physicians and RNs. Although caring is viewed as profession‐neutral, the focus is on RNs in prehospital care because they have primary responsibility for both caring and nursing. Nursing is understood as a professionalized form of caring [3], central to RN education and practice. While the synthesis highlights the RN role, EMS is inherently inter‐ and multi‐professional; thus, the conceptual framework is applicable across prehospital care professions and supports a holistic, integrated approach to patient care.

## 2 Background

Prehospital emergency care is largely shaped by a biomedical perspective emphasizing diseases, clinical signs, diagnoses and medical interventions. As a result, research is predominantly rooted in medical science and centred on emergency treatment for conditions such as cardiac arrest [4], trauma [5], myocardial infarction [6], sepsis [7], resuscitation [8] and stroke [9]. This orientation has produced a care model governed by medically based guidelines and triage systems [10]. Yet patients in emergency settings also experience profound vulnerability, exposure and powerlessness [11], and consistently stress the need for human presence, existential support [12, 13, 14, 15] and being taken seriously [16]. The patient–healthcare professional encounter is considered fundamental [17], and understanding the patient's lifeworld is essential for recognizing the person behind the illness [18]. Despite this, patient perspectives remain underrepresented in prehospital research [19], where studies tend to prioritize healthcare professionals' views on caring [20, 21, 22, 23, 24, 25]. Overall, caring‐related literature in this field is limited compared to other healthcare contexts.

Prehospital research has also focused heavily on patient safety [26, 27], non‐conveyance decision‐making [28, 29, 30], handover communication [31] and quality measurement [32, 33], contributing to practice shaped by logistics, efficiency and safety protocols.

In summary, significant gaps remain regarding interpersonal and ethical dimensions of prehospital emergency care from a caring science perspective [19]. Given its importance across clinical practice, education, research and administration [19, 34], there is a clear need for contextually grounded caring conceptualisations, models and theories. Such frameworks can strengthen caring relationships and illuminate outcomes beyond medical treatment. The aim of this paper is therefore to develop a concept synthesis [35] rooted in a caring science paradigm within the prehospital emergency care context, clarifying the nature of caring in this setting with emphasis on alleviating patient suffering. While caring can be understood as profession‐neutral, this aim underscores its relevance across ambulance healthcare professionals. In this context, nurses hold a particular responsibility to integrate ethical and relational dimensions alongside biomedical perspectives, making a clearly articulated caring science framework essential to prevent biomedical dominance.

## 3 Theoretical Framework

The theoretical framework aims to reveal the person experiencing the illness by adopting an ontological approach based on three ontological concepts [36]. They are grounded in caring science, understood as a human science with multi‐professional relevance [37]. Within this framework, the human being is viewed as a non‐dualistic, irreducible unity of body, soul and spirit; an integrated whole exceeding the sum of its parts [38]. The work is guided by the four metaparadigm assumptions: *the human being*, *health*, *caring* and *environment* [34]. Building on these, the concept synthesis is developed based on three interrelated core concepts within caring science: *the suffering human being* [39], *the caring relationship* [37] and *the caring environment* [40]. Together, these form the theoretical foundation of the synthesis presented in this paper [41].

### 3.1 The Suffering Human Being

The caring science scholar and philosopher Katie Eriksson [39] argues that suffering must be understood holistically, encompassing bodily, psychological, spiritual and existential dimensions as an inherent part of human life and relationships. Bodily suffering is inseparable from psychological and spiritual suffering, meaning illness and its symptoms may also manifest as mental or existential distress. From this perspective, care‐related suffering can arise alongside medical treatment and interventions. A central form of such suffering is the violation of patient dignity, which, while common, is however deeply serious, as it represents a loss of identity and humanity [39]. Approaching care with conscious or unconscious prejudice therefore threatens not only patient dignity but also the integrity of the caring relationship [39].

### 3.2 The Caring Relationship

The caring relationship is grounded in the understanding that human existence is inherently relational, shaped through intersubjective co‐existence in a shared world [18]. Human beings depend on one another, making caring an act of mutuality in which both individuals are simultaneously agents and recipients [42]. This mutuality becomes visible when the patient is recognized and affirmed as a subject, with trust forming the ethical foundation of caring [43]. Such trust creates an ethical demand for the ambulance healthcare professional to assume responsibility, arising from the patient's vulnerability [44]. However, this responsibility must not become unlimited or paternalistic, as this would compromise the patient's agency and erode trust.

The caring relationship is also marked by an inherent asymmetry of power—an ontological condition that grants the ambulance healthcare professional authority. This power must be acknowledged and ethically managed to safeguard the patient's integrity [45]. Caring relationships substantially contribute to patient well‐being; without them, patients risk being objectified and reduced to ‘clinical cases’ rather than recognized as human beings with dignity. Thus, trust—both the patient's trust in the healthcare professional and the healthcare professional's reciprocal trust in the patient—remains the cornerstone of caring relationships [46, 47, 48], whereas distrust and bias may reinforce asymmetry and enable paternalistic exercise of professional power [49].

### 3.3 The Caring Environment

Within caring science, ‘environment’ encompasses both internal and external structures, including family, social networks, professional communities and the broader physical surroundings [50]. At the same time, the caring environment is shaped by the care encounter that unfolds within the patient's personal space.

A place is understood as a space inhabited by the person—one that influences their internal and external possibilities and their perception of the world. It thus becomes “the existential address of the human being in the world” (p. 93) [51]. Both physical and psychosocial dimensions of healthcare environments are essential in caring, regardless of context [52]. Within these environments, ‘rooms’, as conceptual and physical spaces, play a significant role. Understanding the healthcare environment therefore involves considering concepts such as midpoint, atmosphere, surroundings and relationships, all of which shape the overall experience of caring [53]. In the prehospital emergency care context, the caring environment can be understood as a liminal space, situated between the patient's everyday world and the healthcare setting. It is a space where vulnerability, uncertainty, hope and expectation often coexist as patients transition from perceived danger toward safety and care. The quality of this space is shaped not only by its physical surroundings but also by the caring relationship established during the encounter.

## 4 Development of the Concept Synthesis

The concept synthesis was developed on the premise that the theoretical framework should serve as a foundation. A combined deductive–inductive approach was used to articulate the concepts of the theoretical framework (i.e., ‘The suffering human being’, ‘The caring relationship’ and ‘The caring environment’) as clinical‐theoretical concepts within the prehospital context [34]. This culminated in the acronym STOP, representing *acute Suffering* (S), the *act of Togetherness* (T) and the *On‐scene space* (O) in *Prehospital emergency care* (P), together forming the STOP‐synthesis.

In line with Walker and Avant [35], the aim was not to establish fixed or singular definitions, but to clarify and extend these concepts by identifying new dimensions within the context of prehospital emergency care. The study adopts a descriptive and exploratory approach as an initial step in theory development, focusing on the refinement and contextualization of concepts rather than on their philosophical justification. This work should thus be regarded as an early stage in theory building. Rather than aiming to develop a theory, the purpose is to use concept synthesis to clarify, extend and contextualize existing concepts in order to generate new theoretical insights. By identifying and articulating new dimensions of these concepts, the study seeks to enhance their relevance for both research and clinical practice. According to Walker and Avant [35], several approaches to concept synthesis exist, one of which involves identifying new dimensions of existing concepts. Accordingly, the previously well‐defined and well‐studied concepts presented in the ‘Theoretical framework’ were treated as concepts in need of further development in relation to the prehospital emergency care context.

The synthesis emerged through a dialogical process integrating the theoretical framework, the authors' clinical experiences and existing literature [35, 54]. Deduction guided the construction of the theoretical basis, while induction informed the interpretation of empirical experiences and research (Figure 1), resulting in an abductive overall approach [35]. The work followed a hermeneutic circular methodology [54], characterized by continuous movement between theory and the emerging synthesis, shaped through collaborative and critical dialogue among all authors [54]. The synthesis was conducted as an iterative and non‐linear process, in accordance with Walker and Avant's approach to concept synthesis [35]. The work unfolded through interrelated phases that were revisited and refined throughout the process. Initially, the theoretical framework was adopted and interpreted in relation to the authors' clinical and pedagogical experiences. Three of the authors have between 20 and 40 years of clinical experience and research in prehospital emergency care and extensive involvement in university‐level education of ambulance nurses, while the fourth author has substantial experience in such education. This combined experience supported the identification of new dimensions of the concepts and their implications for theory and practice. While the relative homogeneity among the authors may have contributed to a shared pre‐understanding—both a strength and a limitation—some variation exists. One author contributes primarily a pedagogical perspective, while the others bring both clinical and pedagogical experience, providing sufficient diversity for a critical analysis. The authors' experience was complemented by a review of relevant literature, informing and refining the ongoing interpretations. Through this iterative interplay between theory, experience and literature, concepts were derived, analysed and synthesized, culminating in the final formulation of the STOP synthesis.

**FIGURE 1:**
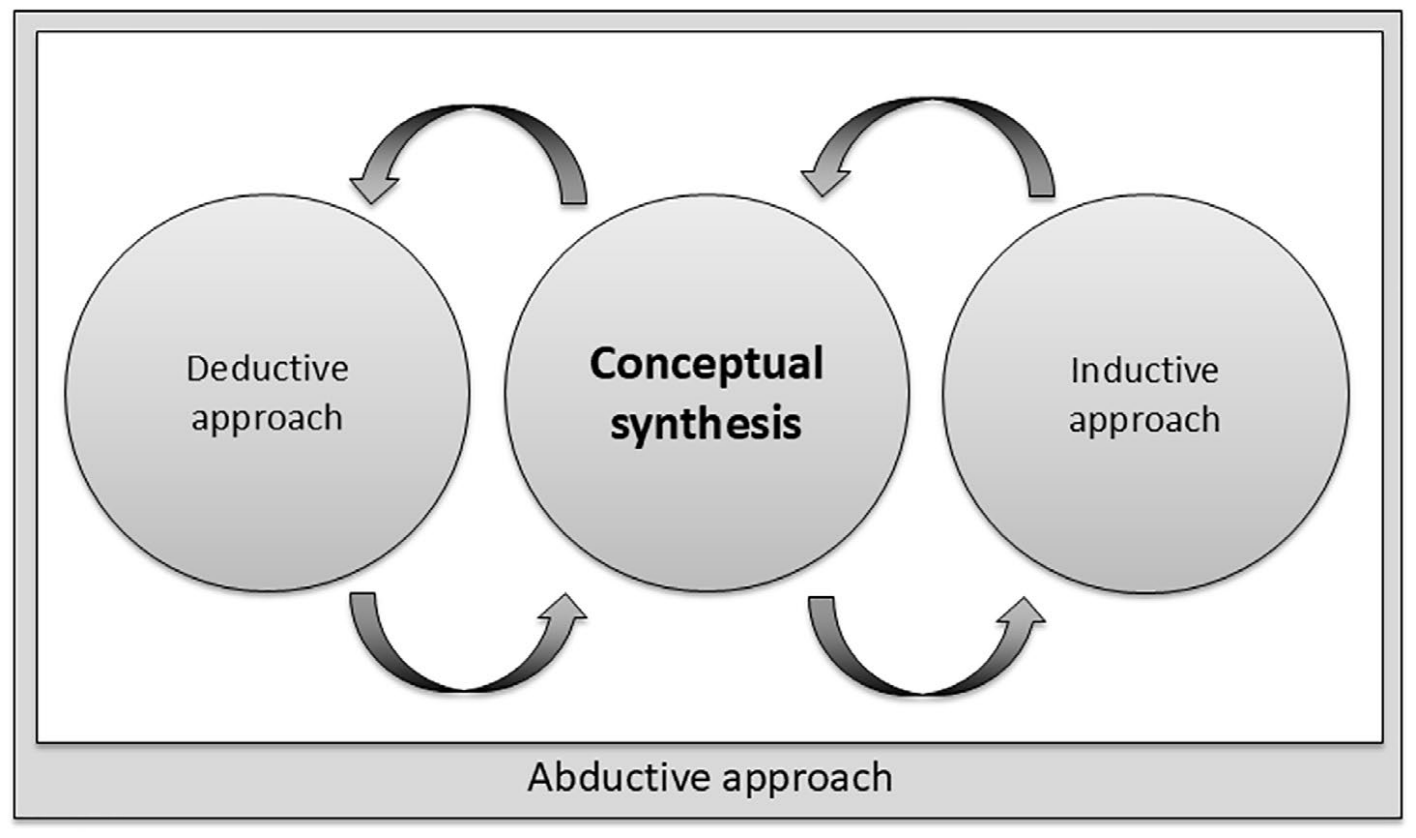
Development of the STOP‐synthesis.

## 5 The STOP‐Synthesis

The STOP‐synthesis reconceptualizes acute prehospital situations through a caring science perspective by positioning the patient's perceived suffering as the starting point. It consists of three interdependent dimensions—*acute Suffering* (S), *act of Togetherness* (T) and *On‐scene caring space* (O)—all situated within *Prehospital emergency care* (P). These dimensions can be envisioned as concentric circles surrounding the patient's subjective experience of *acute suffering*, which forms both the ethical imperative and clinical point of departure.

The *act of togetherness* begins when ambulance healthcare professionals arrive, encounter the patient and establish a caring, trust‐based relationship—becoming the primary pathway for alleviating suffering. Surrounding this is the *on‐scene space*, comprising the physical, social and relational environment, including interprofessional collaboration, significant others and bystanders. This space shapes the conditions for care and must be actively managed to support the alleviation of suffering. It also marks the boundary of the healthcare professional's operational field, requiring adaptation, situational control and the creation of conducive conditions for care. Importantly, the on‐scene space is not only physical but also relational and existential, shaped by intersubjectivity and the dynamics of the encounter.

The three dimensions continuously influence one another. *Acute suffering* may intensify due to environmental threats (e.g., weather, violence, public exposure), and ethical values may be challenged when dignity is jeopardized. Likewise, a chaotic or unsafe *on‐scene space* can hinder the *act of togetherness* and complicate the establishment of a caring relationship.

In summary, the patient's *acute suffering* is alleviated through the act of togetherness, situated within and shaped by the on‐scene space. Addressing the patient's vulnerability and powerlessness requires clinical competence, ethical sensitivity and the ability to foster trust—grounded in an understanding of human interdependence.

### 5.1 Acute Suffering (S)

Acute suffering encompasses physical, care‐related and existential dimensions. It may arise suddenly, without warning, or develop from signs of impending distress. While terms such as *emergency*, *urgent* and *severe* help contextualize its nature, acute suffering is fundamentally subjective and unique to each patient. It may result from sudden illness or trauma, or from deterioration of a chronic condition. In life‐threatening situations, patients often experience fear of death and a profound shift from relative health to vulnerability. Emotions such as fear, anger, despair, powerlessness and physical pain are common elements of acute suffering [39]. One example of this suffering is pain, which is a prevalent symptom in prehospital emergency care; however, it is often underrecognized, underestimated and undertreated. As pain is both a subjective and an objective experience, suffering should be understood as encompassing both biological and existential dimensions [55]. Importantly, the patient's needs are not tied to a single type of suffering; rather, suffering is multifaceted and individually experienced. In the STOP‐synthesis, acute suffering is viewed as a genuine, subjective phenomenon [39] and thus becomes the ethical and clinical starting point for caring interventions.

When patients recognize that their own resources are insufficient, they may be forced to relinquish independence [15, 56, 57]. Many still try to cope alone—even during serious illness—to maintain control, often denying their need for help [46, 58]. The distinction between acute and non‐acute illness is therefore linked to the patient's perceived ability to manage the situation. Dependence becomes explicit when suffering becomes unbearable and visible [59]. In such situations, significant others may also experience acute suffering, becoming ‘co‐patients’ [60, 61, 62]. This marks a moment of surrender, in which the individual entrusts their care to the healthcare professionals, temporarily relinquishing autonomy while relying on their competence and ethical integrity [15]. This surrender is an existential act reflecting the exhaustion of personal resources [15, 59]. At this breaking point, suffering becomes acute and the patient's autonomy may be overridden [23], resulting in existential distress—or, conversely, relief in handing over responsibility.

Acute suffering is therefore deeply tied to subjectivity and strongly present in acute illness, injury or trauma. In these moments, autonomy and dignity are at risk, creating profound dependence on healthcare professionals and potentially evoking humiliation, shame or guilt [39]. The prehospital environment further intensifies vulnerability and exposes patients to threats against their values—especially autonomy and dignity—linked closely to the concept of the on‐scene space. Ultimately, acute suffering must be understood as a unique, subjective experience in which existential suffering, anxiety and reflections on life, death and the future are inseparable from the physical experience of acute illness.

For example, a patient with acute chest pain may primarily express fear of dying or concern for their family. Attending to such experiences may help healthcare professionals recognize suffering beyond the immediate physical symptoms.

### 5.2 Act of Togetherness (T)

Based on a dialectic understanding of spatial dimensions [51]—centre point, atmosphere, setting, surrounding world and prevailing conditions—the patient occupies the central position in the ambulance healthcare professionals' perspective. This is contextually unique, as healthcare professionals often meet previously unknown patients who have managed their illness alone before help arrives. Healthcare professionals must rapidly assess their condition, determine stability and decide on appropriate care, often without immediate medical oversight. In this setting, togetherness—a collaborative effort involving the patient and, when relevant, for example, significant others, firefighters or police—is essential for assessment, care and decision‐making [63, 64].

The caring atmosphere is co‐created through relationships among patients, healthcare professionals and others, forming a climate that embodies the act of togetherness. Initially, this appears as a mutual caring act in which the healthcare professionals and the patient form a team [64]. Although they contribute from different positions, they are interdependent in creating conditions for continued care and establishing trust [64]. For the patient, this marks a shift from a solitary struggle to a shared effort to alleviate suffering [59]. The act of togetherness is rooted in mutual trust—patients must trust healthcare professionals, and healthcare professionals must honour that trust [43]. Trust depends both on interpersonal interaction and the healthcare professional's role as a representative of a respected professional institution [65]. Trust may be challenged when patients have difficulty participating in their care (e.g., due to intoxication or cognitive impairment) or when they pose a physical threat to staff, thereby challenging the act of togetherness.

Power asymmetry, patient vulnerability and situational uncertainty inherently challenge trust. Ambulance healthcare professionals are perceived as authoritative figures due to their uniforms and societal role [66]. Thus, the act of togetherness becomes an ethical endeavour shaped by inequality and the conditions of the prehospital setting. Patients may accept reduced autonomy, allowing the healthcare professionals to guide care. However, sustaining trust depends on how the healthcare professionals ethically manage their legitimate power, acting with awareness of the unequal distribution of authority [43]. The act of togetherness also involves advocacy: healthcare professionals must represent patients who cannot speak for themselves while supporting autonomy when patients are capable of self‐expression.

Furthermore, togetherness extends to interprofessional collaboration with dynamic, often ad hoc teams that may include significant others [63, 67, 68]. Healthcare professionals must coordinate with other healthcare providers, social workers, nursing home staff, police and firefighters when needed. Effective collaboration requires clearly defined roles to address the patient's acute suffering. Ultimately, the act of togetherness is dynamic, situationally responsive and transcends formal professional boundaries [68].

For example, togetherness may be fostered through attentive listening and presence, confirming the patient's experience and establishing trust in the midst of uncertainty, for example, while experiencing severe acute chest pain.

### 5.3 On‐Scene Space (O)

The on‐scene space in prehospital care is both a prerequisite for care and an integral part of the caring process. Conceptualized through dimensions such as centre point, atmosphere, setting, surrounding world and prevailing conditions, it is simultaneously a physical and functional site for care and a relational, intersubjective environment where meaning is co‐created—forming a caring environment [69]. The on‐scene space cannot be separated from the act of togetherness, but understanding their interaction requires a conceptual grounding.

This space is inherently dynamic. Prehospital care often unfolds in chaotic, hazardous and unpredictable environments [70] such as ambulance vehicles, homes, public areas or streets. Ambulance healthcare professionals must accept the location where the patient is found and adapt themselves and the caring space to prevailing conditions [64]. The *setting* consists of the immediate physical objects and people present, while the *surrounding world* refers to broader contextual factors that still influence the caring space. These dimensions are intertwined with the central focus: the patient and their acute suffering.

Only the ambulance vehicle is designed for care; most prehospital ‘rooms’ must be created in real time. This makes the on‐scene space unique, marked by variability and limited control for the healthcare professionals. Each situation must be arranged according to its conditions [64]. The patient's home may be familiar and secure, while public or accidental locations may be unsafe and uncertain. Thus, a functional caring space must be actively constructed. Although the on‐scene space may appear static and objective, it is also fluid and adaptable. The healthcare professionals accept the ‘given’ space but also reshape it—for instance, using light or heat in public settings to protect dignity and comfort.

Providing care in public spaces adds challenges, as bystanders may witness the situation. This exposure can threaten patient dignity [20], as values, judgements and actions from others may intrude. Safeguarding dignity requires protecting personal space and attending to vulnerability and uniqueness. Healthcare professionals must create a caring space that upholds dignity and supports a trustworthy act of togetherness—essential for accurate assessment and meaningful caring relationships [71].

For example, the on‐scene space may be actively managed by reducing unnecessary exposure, creating privacy and arranging the environment in ways that support dignity and a caring encounter. For instance, when caring for a patient with chest pain in a public place.

## 6 Reflections

The methodological development of the STOP synthesis was guided by Walker and Avant's [35] approach to concept synthesis, enabling the exploration of new dimensions of established caring science concepts within the prehospital emergency care context. The process was iterative and integrative, combining theoretical perspectives, clinical and pedagogical experience, and empirical research to refine and contextualize existing concepts. The aim was not to generate new concepts, which may be seen as a limitation. However, the work builds on a well‐established theoretical foundation developed over decades, contributing through contextual reinterpretation rather than conceptual innovation. While this may limit its contribution to novel theory development, it strengthens coherence and credibility by grounding the synthesis in an established body of knowledge.

Thus, the STOP synthesis should be understood as an initial, exploratory step that refines existing concepts and provides a basis for continued theory development in prehospital emergency care. Caring science aspects in prehospital emergency care remain insufficiently explored and theoretically under‐synthesized [19]. The STOP‐synthesis is supported by research showing patients' insecurity about seeking ambulance care [46, 56] and the need for professional intervention when suffering becomes overwhelming [13, 15, 16]. Violations of dignity may occur when healthcare professionals adopt dismissive attitudes [56], yet prehospital emergency care can also facilitate a caring relationship—an *act of togetherness* [15]—emphasizing the need for patients and significant others to feel taken seriously [16, 62].

Although ambulance nurses are expected to provide expertise grounded in caring science, educational curricula place limited emphasis on this area [72], partly due to scarce contextual literature [19]. The STOP‐synthesis supports a holistic approach by integrating caring science into a medically dominated field, also relevant for other prehospital professions focused on emergency medicine, trauma and disaster response [33, 73]. It thus provides theoretical support for both academic and clinical training. The STOP‐synthesis suggests that prehospital assessment can be understood as extending beyond the identification of symptoms, diagnoses and treatment needs. By also attending to the patient's acute suffering, the possibilities for establishing togetherness and the influence of the on‐scene space, healthcare professionals may gain a more comprehensive understanding of the situation and the caring needs that emerge within it. In this way, the synthesis may contribute to a caring science perspective on assessment and decision‐making in prehospital emergency care.

Within EMS, research priorities have shifted from medical to organizational issues such as coordination and differentiation [30, 33, 74]. Despite these developments, ethical and interpersonal dimensions from a caring science perspective remain underrepresented. The STOP‐synthesis offers a theoretical foundation to inform future research agendas grounded in caring science.

This concept synthesis has a twofold purpose: to provide an initial structure of clinically contextualized concepts grounded in established caring science, offering a complementary perspective to the prevailing biomedical focus, and to serve as a first step toward further theoretical development in prehospital emergency care, where a conceptual framework is currently lacking. While caring can be understood as profession‐neutral, the STOP synthesis is relevant across multiple professional groups within ambulance services. However, nurses bear a particular responsibility to balance ethical and relational dimensions informed by caring science alongside biomedical perspectives. A well‐articulated caring science framework is therefore essential to prevent biomedical dominance. At the same time, situations such as cardiopulmonary resuscitation necessitate a reductionist view of the body; yet even then, the interplay between biological and existential dimensions remains inherent [68]. This responsibility is particularly salient in nurse‐led prehospital emergency care systems, such as those in Sweden and Finland. However, empirical studies, from the perspectives of patients, significant others and professionals, are needed to examine the practical implications of the STOP‐synthesis and its core concepts—*acute Suffering*, the *act of Togetherness* and the *On‐scene space*. Concept analyses and deductive studies involving patients, significant others and healthcare professionals can deepen the concept synthesis and support further theory development.

Together with ongoing work, the STOP‐synthesis advances caring and nursing science within the still underdefined prehospital context. Regardless of professional background, this perspective is essential for supporting a holistic and human‐centred prehospital practice.

## 7 Conclusions

Using the STOP‐sign metaphor, this synthesis underscores the need to pause in prehospital emergency care to reflect on the patient as a multidimensional human being and to critically examine the knowledge base guiding care. It promotes a shift toward a caring science paradigm focused on the human experience of suffering and care.

Prehospital emergency care must expand beyond a biomedical lens to encompass a broader theoretical understanding of suffering and caring. The STOP‐synthesis integrates four interrelated concepts: *acute Suffering (S)*, the *act of Togetherness (T)*, the *On‐scene space (O)* and the *Prehospital emergency care (P)* context.

- *Acute suffering* involves illness‐related, care‐related and existential dimensions that often arise unexpectedly.
- The *act of togetherness* denotes the relational process through which suffering is alleviated, characterized by mutual dependence that supports a transition from isolation to shared care.
- *On‐scene space* reflects the unpredictable environments shaping prehospital care, functioning as both a condition for and expression of caring.
- These concepts operate within the broader *prehospital emergency care* context, requiring adaptability, ethical sensitivity and human‐centred commitment.

Overall, the STOP‐synthesis supports sustainable and ethical development in prehospital emergency care by positioning the unique human being at the centre of the caring encounter. It provides a theoretical basis for future research, education and clinical practice aligned with caring science, promoting a holistic understanding of care in urgent and complex situations. In a context often characterized by a ‘find‐it, fix‐it’ modus operandi, this synthesis further underscores the importance of ethical and relational dimensions as integral to high‐quality care, ensuring that the person is not reduced to a solely biomedical concern.

## Author Contributions

The authors contributed equally to the planning, execution and writing of this paper, with the first and last authors serving as project leaders and holding overall responsibility for the process.

## Funding

This paper was partly funded by the Alice Lindström's study foundation, The Swedish Society of Nursing.

## Ethics Statement

Ethical approval or any form of ethical review or assessment was not applicable for this theoretical paper, as it did not involve empirical data, human participants or interventions requiring ethical clearance.

## Conflicts of Interest

The authors declare no conflicts of interest.

## Data Availability

Data sharing not applicable to this article as no datasets were generated or analysed during the current study.
